# Linear Interval Approximation of Sensor Characteristics with Inflection Points

**DOI:** 10.3390/s23062933

**Published:** 2023-03-08

**Authors:** Marin B. Marinov, Nikolay Nikolov, Slav Dimitrov, Borislav Ganev, Georgi T. Nikolov, Yana Stoyanova, Todor Todorov, Lachezar Kochev

**Affiliations:** 1 Faculty of Electronic Engineering and Technologies, Technical University of Sofia, 1756 Sofia, Bulgaria; 2 Faculty of Industrial Technology, Technical University of Sofia, 1756 Sofia, Bulgaria

**Keywords:** approximation, graphical programming, Internet of Things, linearization techniques, measurement errors, smart sensors, sensor accuracy, thermocouples

## Abstract

The popularity of smart sensors and the Internet of Things (IoT) is growing in various fields and applications. Both collect and transfer data to networks. However, due to limited resources, deploying IoT in real-world applications can be challenging. Most of the algorithmic solutions proposed so far to address these challenges were based on linear interval approximations and were developed for resource-constrained microcontroller architectures, i.e., they need buffering of the sensor data and either have a runtime dependency on the segment length or require the sensor inverse response to be analytically known in advance. Our present work proposed a new algorithm for the piecewise-linear approximation of differentiable sensor characteristics with varying algebraic curvature, maintaining the low fixed computational complexity as well as reduced memory requirements, as demonstrated in a test concerning the linearization of the inverse sensor characteristic of type K thermocouple. As before, our error-minimization approach solved the two problems of finding the inverse sensor characteristic and its linearization simultaneously while minimizing the number of points needed to support the characteristic.

## 1. Introduction (and Motivation)

### 1.1. Resource-Constrained Smart Sensor Devices and IoT

In recent years, there has been a growing interest in smart low-cost sensor technologies and IoT [1]. Many very promising developments were made in the field of both sensor technology and wireless communications. Research in the field of smart sensors (SS) and the Internet of Things (IoT) continues to grow [2,3]. Advances in microcontrollers and developments in the Internet have enabled the industry to create smart devices that efficiently integrate sophisticated sensing and communication functions, with primary signal-processing algorithms.

A key distinguishing feature of low-cost smart sensors and IoT devices is their *limited resources*. Typical self-powered smart sensors and IoT devices include two main resource groups. 

*Hardware resources:* for data storing and computing, communication, and power. 

Because IoT devices are battery-powered and use low-power processors, they cannot accommodate algorithms that require a large amount of computing power. Furthermore, IoT devices have limited memory compared to regular digital systems, requiring the use of lightweight mobile software and operating systems. As a result, the algorithms must be designed in a way that is efficient in terms of memory usage [4,5].

*Software resources:* operating systems, system software, and applications. The adaptive allocation of these resources is critical in most applications [6,7]. The remote reprogramming of devices is not always an option as the operating system may not be capable of accepting and integrating new codes.

### 1.2. The Main Error Components of Smart Sensors and IoT Devices

**Figure 1:**
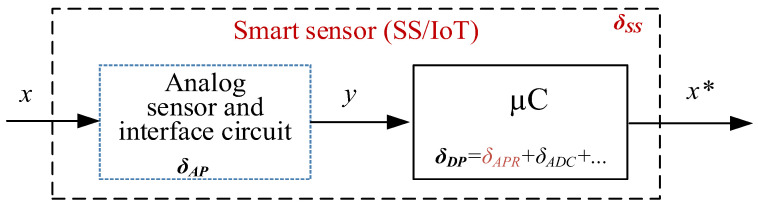
Block diagram and basic error components of smart sensor and IoT devices (*x*—input quantity, x*—microcontroller output).

As already stated, the main objective of this study was to seek opportunities to allocate the limited resources of networked sensors and IoT adaptively and, in particular, to limit the amount of memory required without compromising the accuracy requirements of the devices. The structure of a smart sensor or an IoT device and its main components are shown in Figure 1. 

For the devices to meet the desired accuracy standards, the major sources of error must be considered. These can be divided into $\delta_{AP}$ which are sourced from the analog portion and $\delta_{DP}$ which are sourced from the digital part. Further information can be found in [1] and [8].

Here, $\delta_{AP}$ is the relative compound error of the analog sensor and sensor interface, $\delta_{DP}$ is the relative compound error of the digital part, $\delta_{AP}=\frac{\Delta^{x}}{x_{FS}}$ is the relative approximation error, $\Delta^{x}\;$ is the predefined absolute approximation error; $x_{FS}$ is the full scale (FS) of the microcontroller output value x*; and $\delta_{ADC}$ is the relative error of the analog-to-digital conversion. Given a serial device structure, the total error budget of the system can be determined as follows:

$$\delta_{SS}=\sqrt{\delta_{AP}^{2}+\delta_{DP}^{2}}.$$

$\delta_{ADC}$ and $\delta_{APR}$ are the main error components of the $\delta_{DP}$ digital part. Once the analog sensor and the analog-to-digital converter (ADC) are selected in the device implementation, the error levels of $\delta_{AS}$ and $\delta_{ADC}$ cannot be affected. ADCs with high resolution are most commonly used in the implementation of smart sensors and IoT; therefore, the $\delta_{ADC}$ component is often negligible. This makes $\delta_{APR}$ an essential component by which the overall error rates can be controlled. To achieve typical accuracy levels in device design, where $\delta_{SS}\approx \delta_{AS}$, it is usually sufficient to achieve the condition $\delta_{AS}>\left(3÷10\right)\delta_{DP}$.

Published lookup tables, which are used in many applications, are often limited in the number of digits and this can be an additional source of rounding errors [8].

### 1.3. Sensor Characteristics Linearization Approaches

Linearization is an important part of the initial processing of sensor inputs. Nonlinearities in sensors can be reduced by either electronic linearization circuits or algorithms [9,10]. Linearization techniques can be divided into three main categories:

- Analog hardware linearization schemes;
- Linearization algorithms;
- Mixed hardware and software-based approaches [11].

Analog hardware linearization methods are typically performed by connecting an analog scheme between the sensor and the ADC [12].

In [13], the focus is on the cold-end compensation of thermocouples used in room temperature measurement applications. In [14], an approach to linearize the characteristics of a K-type thermocouple and thermistor used for its temperature compensation was shown. A reduction in the nonlinearity of about 100 times for the thermocouple and from 84% to 0.27% for the thermistor was demonstrated. 

Software linearization techniques necessitate the deployment of (micro)computers or digital signal processors (DSPs) with powerful processing capabilities [15,16]. Implementing these techniques on low-cost controllers that only have the capacity for limited computational tasks is very difficult due to the limited resources of the controller. Different software linearization methods were analyzed in the literature. One of the most frequently used methods was the lookup table (LUT)-based linearization, which can be implemented easily on any microcontroller [11,17].

This research built upon the technique outlined in [1,18], which was used to adaptively linearize sensor characteristics, make the design simpler, and improve the measurement accuracy of sensors and IoT devices that are resource-limited.

The identification of the inverse transfer function is often complicated due to the difficulty in selecting the correct analytic form of the function and the constraints in its parameterization. This can lead to inaccurate sensor responses, so it should be avoided. Generally, the scalar inverse sensor transfer function is modeled using a nonlinear regression model (e.g., polynomial, exponential, etc.) which is determined by minimizing the least squares error on a statistically representative set of data [19,20].

In [21], the use of a simple model based on a three-layer recurrent neural network (RNN) that considers a short history of the immediately preceding predictions (temperatures) together with the current measurement to predict the current output signal was shown. In [22], the use of popular sensor linearization techniques based on the multilinear model approach was shown to be popular due to the simplicity and transparency of local linear models. The paper presented a systematic data-driven approach that used the included angle method to determine optimal linear models. A fuzzy interpolation technique was then used to combine the linear models.

Linearization approaches can be also regarded as shape-preserving dimensionality-reducing methods [23]. In such methods, the inverse sensor characteristic is mapped into a polygonal shape, by using either distance minimizing embedding technique, or, whenever permissible, by range and accuracy requirements—a non-negative matrix factorization technique, as suggested in [24].

A general approach to reducing the uncertainty problems arising in nonlinear regression identification of sensor feedback can be provided by segmenting its transfer function. 

Essentially, it implements a polygonal approximation of $x=x\left(y\right)$ with approximation error control. The proposed algorithmic control is an important aspect supporting adaptive resource allocation.

Piecewise linear approximation (PLA) for sensor data is a classical approach used in data compression. There are many other data compression approaches, such as piecewise aggregate approximation [25], discrete wavelet transform [26], discrete Fourier transform [27], Chebyshev polynomials [28], etc. However, PLA remains one of the most commonly used approaches for data compression, as claimed in [29,30].

The approach dates back to the middle of the last century but, in recent years, the data compression problem became topical due to the widespread adoption of smart sensors and IoT devices. It was increasingly used wherever data acquisition devices limited local buffer space and communication bandwidth [31].

It is essential that data are compressed due to the restricted resources of data acquisition devices, including memory and communication capabilities. The main criteria for measuring compression quality are the magnitude of the approximation error and the number of line segments.

In optimizing the PLA results, two approaches are commonly used: 

Setting a bound on the error $\Delta^{x}$ and minimizing the number of k segments;

Setting the number of k segments for which to construct a PLA with at most k segments that minimize the error $\Delta^{x}$. 

In the approach investigated in this paper, we set the delta error bound and minimized the PLA size [32].

### 1.4. Piecewise Linear Approximation of Sensor Characteristics with Inflex Points

In [15], an algorithm was formulated to linearize sensor characteristics when the characteristic was a differentiable function. This two-step algorithm involved an iterative process with a given sensor characteristic and a maximum approximation error. The result of this process was a discrete form (a broken line) of the inverse characteristic. 

With a set sensor characteristic in the form $y=y\left(x\right)$ the first step is to determine the inverse sensory characteristic $x=x\left(y\right)$. The second step is related to the approximation of the received discrete inverse sensory characteristic in the species $x_{i}=x_{i}\left(y_{i}\right),i=\bar{1,n}$ with a new feature $x_{j}=x_{j}\left(y_{j}\right),\;\;j=\bar{1,k}$ given the maximum approximation error and minimization of k. An approach to solving the problem was developed in [15] and in [33].

In [1], a novel technique for linearizing the characteristics of sensors, which were represented by differentiable functions with a constant sign of curvature, was proposed. This approach simultaneously solved the issues of locating the inverse sensor characteristic and its linearization, as was exemplified by the resistance–temperature relationship of platinum temperature sensors that followed the Callendar–Van Dusen equation. The approach was characterized by the fact that when the maximum approximation error is set $\Delta^{x}$ in the linearization of the inverse sensing characteristic, the inverse sensing characteristic $x_{i}=x_{i}\left(y_{i}\right),i=\bar{1,n}$ is found directly in linearized form. The advantages of the developed approach are as follows:

- The approach is applied in intervals, and at each subsequent step (each subsequent interval) as a result of analogously solving the task under the new initial conditions, the desired solution is obtained directly, containing, in turn, the initial conditions for the next step;
- The maximum linearization error of the inverse sensor characteristic in all intervals is the same;
- The approach makes it possible to set a different maximum approximation error in each subsequent interval.

The proposed approach was tested in the linearization of the inverse sensor characteristic of Pt100 sensors, where the relationship between the resistance and the temperature was set by employing the Callendar–Van Dusen equation [34,35]. When the maximum approximation error is set $\Delta^{T}=-0.375\;℃$ in the interval $T\in \left[-200\;℃,\;661\;℃\right]$ the reverse sensory characteristic $T\left(R\right)$ (representing a broken line) is described by 11 points.

**Figure 2:**
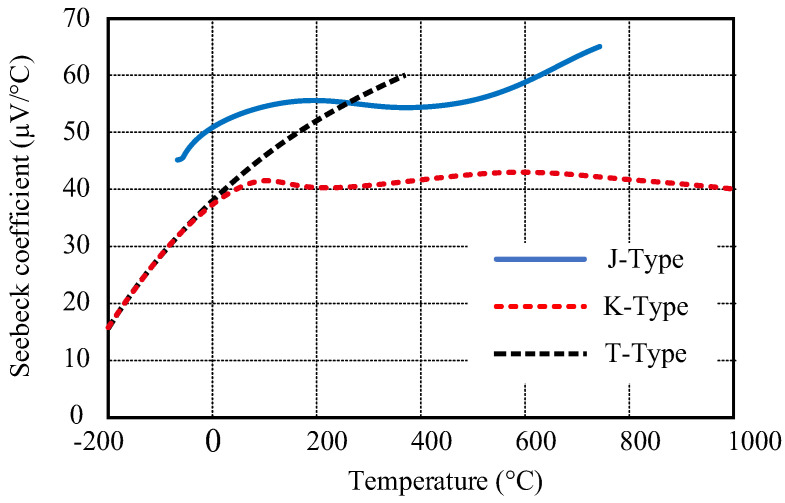
Temperature-dependent sensitivity change of three types of thermocouples (adopted from [36]). K- and J-type thermocouples were chosen for the tests in the study.

Thermocouples are simple and widely used sensor elements for temperature measurement. However, it is not easy to convert the voltage generated by the thermocouple to temperature with high accuracy for several reasons, the main ones being the low levels of useful signal and the non-linear relationship between temperature and voltage. The thermocouples also change their sensitivity depending on the temperature, as seen in Figure 2. This is why they were chosen for the tests in our study.

This approach for approximating sensor characteristics $y=y\left(x\right)$ with linear intervals can be used for features that contain inflection points, where the curvature changes in different subintervals (i.e., “concave” and “convex” functions). An example of this is type K thermocouples [37]. To do this, the approach must be applied separately to each of the subintervals in which the sensory characteristic is “concave” and separately to each of the subintervals in which the sensory characteristic is “convex”. However, this would lead to the elimination of one of the main advantages of the approach developed in [1]—the maximum linearization error of the inverse sensor response in all intervals (except the last one) to be the same.

In the context of the above, this paper was devoted to the development of a generalized approach for linear point-interval approximation of sensor characteristics, representing differentiable functions, with inflection points present. The main advantage of this is that, similar to the approach developed in [1], the tasks of finding the inverse sensor characteristic and its linearization are solved simultaneously.

**Figure 3:**
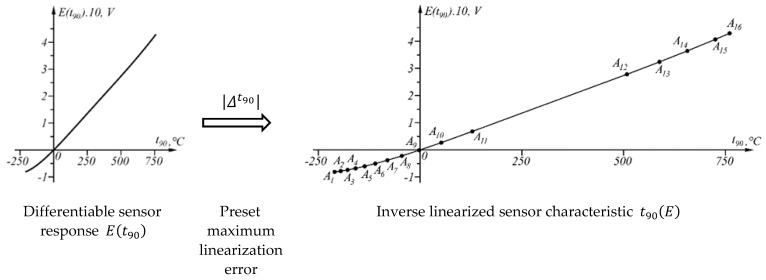
Graphical representation of the most important part of the proposed approach for linearization.

The proposed generalized approach for linear point-interval approximation of sensor characteristics applies to all sensory characteristics, representing differentiable functions. The essence of the approach is illustrated in Figure 3.

The preset maximum linearization error is the only parameter in the generalized approach for linear point-interval approximation of sensor characteristics, on which the number of segments of the broken line (inverse linearized sensor characteristic) depends. A smaller value of the parameter (the maximum linearization error) results in more segments. The particular value of this parameter is determined by the application of the sensor and the accuracy target set.

## 2. The Analytical Frame of the Proposed Approach 

In [1], an approach for the linearization of sensor characteristics representing differentiable functions with an invariant sign of curvature was presented. In the present study, this approach will be further developed for cases where sensory features include inflection points.

Let a differentiable sensor characteristic be given in the form $y=y\left(x\right)$, $x\in \left[x_{A_{i}},x_{B}\right]$, $i=\bar{1,n-1}$. The goal is to obtain the inverse sensor characteristic $x\left(y\right)$ in a linearized form with a set maximum approximation error $\Delta^{x}$.

**Figure 4:**
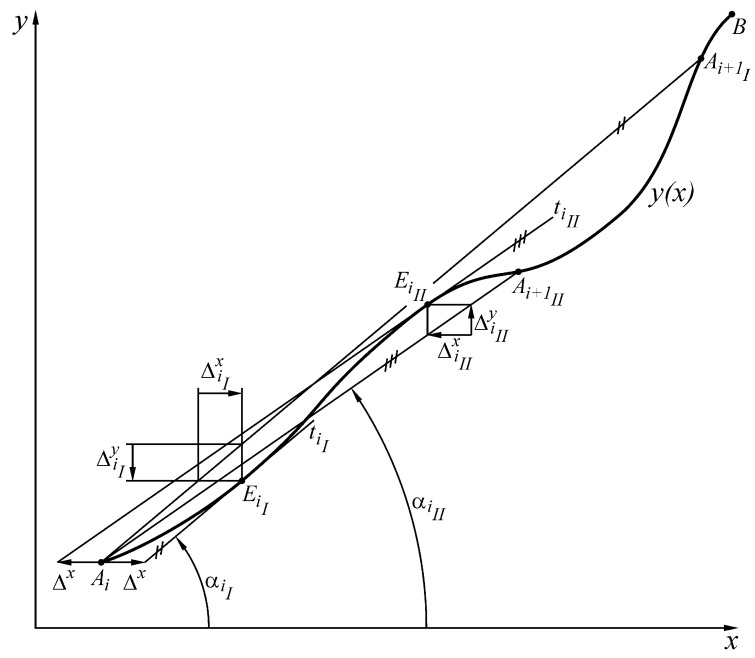
An example of mathematical processing of sensor characteristic.

The approach is based on the following. We are looking for the closest to point $A_{i}$ point $E_{i_{I}}$ from the curve $y=y\left(x\right)$, such that tangent $t_{i_{I}}$ to the curve at point $E_{i_{I}}$ passes through the coordinate point $\left(x_{A_{i}}+\Delta^{x},y_{A_{i}}\right)$ (Figure 4). 

Then, we search for the closest to point $A_{i}$ point $A_{i+1_{I}}$ from curve $y=y\left(x\right)$, being the point of intersection of curve $y=y\left(x\right)$ with the line passing through point $A_{i}$ and parallel to the tangent $t_{i_{I}}$. Analogously, we look for the closest to point $A_{i}$ point $E_{i_{II}}$ from curve $y=y\left(x\right)$, such that the tangent $t_{i_{II}}$ to the curve at point $E_{i_{II}}$ to pass through the coordinate point $\left(x_{A_{i}}-\Delta^{x},y_{A_{i}}\right)$. Then, we search for the closest to point $A_{i}$ point $A_{i+1_{II}}$ from curve $y=y\left(x\right)$, being the point of intersection of curve $y=y\left(x\right)$ with the line passing through point $A_{i}$ and parallel to the tangent $t_{i_{II}}$ (Figure 4). The closer to the point $A_{i}$ of the points $A_{i+1_{I}}$ and $A_{i+1_{II}}$ is the endpoint $A_{i+1}$ of the interval in which the maximum absolute value of the error is $\Delta^{x}\;$when approximating the inverse sensor response $x\left(y\right)$ with a linear dependence (the segment $A_{i}A_{i+1}$).

If after some value of i, the interval $x\in \left[x_{A_{i}},x_{B}\right]$ does not include an inflection point (the function $y=y\left(x\right)$ is “concave” or “convex” in the interval), then one of the two tangents $t_{i_{I}}$ and $t_{i_{II}}$ does not exist, and the approach presented here is practically reduced to the approach presented in [1].

If $x_{A_{i+1}}>x_{B}$, then $A_{i+1}\equiv B$ and the procedure ends, as in the general case in this last interval, the maximum absolute error is less than the set $\Delta^{x}$.

In the general case, when range $x\in \left[x_{A_{i}},x_{B}\right]$ includes an inflection point, the two extremes $\Delta_{i_{I}}^{x}$ and $\Delta_{i_{II}}^{x}$ of error $\Delta_{i}^{x}\left(y\right)$ in the linear approximation of the inverse function $x\left(y\right)$ are determined by (Figure 4):

(1) $$\begin{array}{l} \Delta_{i_{I}}^{x}\left(x_{E_{i_{I}}}\right)=\frac{\Delta_{i_{I}}^{y}\left(x_{E_{i_{I}}}\right)}{-k_{i_{I}}}=x_{E_{i_{I}}}-x_{A_{i}}-\frac{y\left(x_{E_{i_{I}}}\right)-y_{A_{i}}}{k_{i_{I}}};\\ \Delta_{i_{II}}^{x}\left(x_{E_{i_{II}}}\right)=\frac{\Delta_{i_{I}}^{y}\left(x_{E_{i_{II}}}\right)}{-k_{i_{II}}}=x_{E_{i_{II}}}-x_{A_{i}}-\frac{y\left(x_{E_{i_{II}}}\right)-y_{A_{i}}}{k_{i_{II}}}, \end{array}$$

where

$$\begin{array}{l} \frac{\Delta_{i}^{y}\left(x_{E_{i_{I}}}\right)}{\Delta_{i}^{x}\left(x_{E_{i_{I}}}\right)}=-\tan\alpha_{i_{I}}=-k_{i_{I}};\\ \frac{\Delta_{i}^{y}\left(x_{E_{i_{II}}}\right)}{\Delta_{i}^{x}\left(x_{E_{i_{II}}}\right)}=-\tan\alpha_{i_{II}}=-k_{i_{II}}. \end{array}$$

From the extremum necessary condition ${\left[\Delta_{i_{I}}^{y}\left(x_{E_{i_{I}}}\right)\right]}^{\prime }=0$, respectively, ${\left[\Delta_{i_{II}}^{y}\left(x_{E_{i_{II}}}\right)\right]}^{\prime }=0$, is obtained:

(2) $$\begin{array}{l} y^{\prime }\left(x_{E_{i_{I}}}\right)-k_{i_{I}}=0;\\ y^{\prime }\left(x_{E_{i_{II}}}\right)-k_{i_{II}}=0. \end{array}$$

From Equations (1) and (2) follows:

$$\Delta_{i_{I}}^{x}\left(x_{E_{i_{I}}}\right)=\Delta^{x}=x_{E_{i_{I}}}-x_{A_{i}}-\frac{y\left(x_{E_{i_{I}}}\right)-y_{A_{i}}}{k_{i_{I}}}=x_{E_{i_{I}}}-x_{A_{i}}-\frac{y\left(x_{E_{i_{I}}}\right)-y_{A_{i}}}{y^{\prime }\left(x_{E_{i_{I}}}\right)};$$

$$\Delta_{i_{II}}^{x}\left(x_{E_{i_{I}}}\right)=-\Delta^{x}=x_{E_{i_{II}}}-x_{A_{i}}-\frac{y\left(x_{E_{i_{II}}}\right)-y_{A_{i}}}{k_{i_{II}}}=x_{E_{i_{II}}}-x_{A_{i}}-\frac{y\left(x_{E_{i_{II}}}\right)-y_{A_{i}}}{y^{\prime }\left(x_{E_{i_{II}}}\right)}.$$

respectively

(3) $$\begin{array}{l} y^{\prime }\left(x_{E_{i_{I}}}\right)-\frac{y\left(x_{E_{i_{I}}}\right)-y_{A_{i}}}{x_{E_{i_{I}}}-x_{A_{i}}-\Delta^{x}}=0;\\ y^{\prime }\left(x_{E_{i_{II}}}\right)-\frac{y\left(x_{E_{i_{II}}}\right)-y_{A_{i}}}{x_{E_{i_{II}}}-x_{A_{i}}+\Delta^{x}}=0. \end{array}$$

From the last equation, we determine the nearest points $E_{i_{I}}$ and $E_{i_{II}}$ (in case there are more than one) to the point $A_{i}$. 

From the equations

(4) $$\begin{array}{l} y\left(x_{A_{i+1_{I}}}\right)=y_{A_{i}}+y^{\prime }\left(x_{E_{i_{I}}}\right)\left(x_{A_{i+1_{I}}}-x_{A_{i}}\right),\\ y\left(x_{A_{i+1_{II}}}\right)=y_{A_{i}}+y^{\prime }\left(x_{E_{i_{II}}}\right)\left(x_{A_{i+1_{II}}}-x_{A_{i}}\right), \end{array}$$

 $x_{A_{i+1_{I}}}$ and $x_{A_{i+1_{II}}}$ are determined, with the smaller of the two values representing $x_{A_{i+1}}$ (i.e., the point closest to the point $A_{i}$ of the points $A_{i+1_{I}}$ and $A_{i+1_{II}}$ being the endpoint $A_{i+1}$ of the interval).

As a result of applying the approach, the sensory characteristic $y\left(x\right)$ is approximated by the broken line $A_{1}A_{2}A_{3}\ldots A_{n-1}B\equiv A_{n}$, $A_{i}\left(x_{A_{i}},y_{A_{i}}\right)$, $i=\bar{1,n-1}$, $A_{n}\left(x_{B},y_{B}\right)$.

The inverse sensor characteristic $x\left(y\right)$ in coordinate system yx is approximated by the broken line $A_{1}A_{2}A_{3}\ldots A_{n-1}B\equiv A_{n}$, $A_{i}\left(y_{A_{i}},x_{A_{i}}\right)$, $i=\bar{1,n-1}$, $A_{n}\left(y_{B},x_{B}\right)$, as

(5) $$\begin{array}{l} x\left(y\right)=x_{A_{i}}+\left(y-y_{A_{i}}\right)\frac{x_{A_{i+1}}-x_{A_{i}}}{y_{A_{i+1}}-y_{A_{i}}},\\ y\in \left[y_{A_{i}},y_{A_{i+1}}\right],\;i=\bar{1,n-1}. \end{array}$$

In the general case, in all intervals, except for the last one, the maximum errors of the approximation are equal and equal to the specified maximum error $\Delta^{x}$. The error $\Delta_{i}^{x}\left(x\right)$ in each of the intervals is determined by

(6) $$\begin{array}{l} \Delta_{i}^{x}\left(x\right)=x-x_{A_{i}}-\left(y\left(x\right)-y_{A_{i}}\right)\frac{x_{A_{i+1}}-x_{A_{i}}}{y_{A_{i+1}}-y_{A_{i}}},\\ x\in \left[x_{A_{i}},x_{A_{i+1}}\right],\;i=\bar{1,n-1}. \end{array}$$

The error $\Delta_{i}^{x}\left(y\right)$ can also be determined from Equation (6), because the function $y=y\left(x\right)$ is strictly monotonic (at any value of x matches a single corresponding value of $y\left(x\right)$)

(7) $$\begin{array}{l} \Delta_{i}^{x}\left[y\left(x\right)\right]=\Delta_{i}^{x}\left(x\right),\\ x\in \left[x_{A_{i}},x_{A_{i+1}}\right],\;i=\bar{1,n-1}. \end{array}$$

## 3. Linearization of the Inverse Sensor Characteristic of Type K and Type J Thermocouples 

### 3.1. Type K Segmentation in the Temperature Range $t_{90}\in \left[0,1371{.655\;}^{\circ }C\right]$

The thermoelectric voltage E in microvolts, as a function of temperature $t_{90}$ in degrees Celsius, in the range $t_{90}\in \left[0,\;1372{\;}^{\circ }C\right]$, is defined by:

$$E\left(t_{90}\right)=\overset{9}{\underset{i=0}{\sum }}c_{i}\;{\left(t_{90}\right)}^{i}+\alpha_{0}e^{\alpha_{1}{\left(t_{90}-126.9686\right)}^{2}},$$

where the coefficients $c_{i}$, $\alpha_{0}$ and $\alpha_{1}$ are specified in NIST ITS-90 [37].

**Figure 5:**
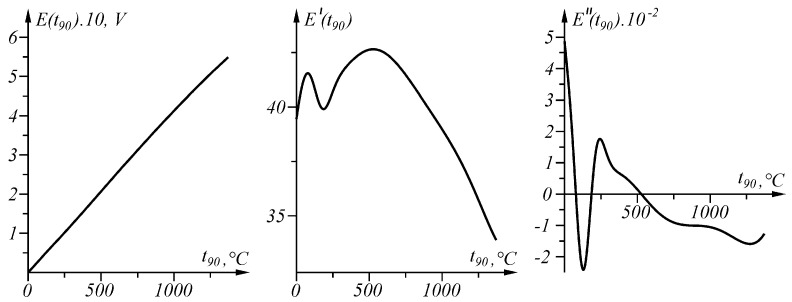
Functions E(t_90), its first derivative E’ (t_90) and second derivative E″ (t_90) in interval t_90 ∈ [0, 1372 °C].

With the help of the proposed approach, the problem of interval linearization of the inverse characteristic of the sensory characteristic in question, including inflection points, will be solved. The graphs of the function $E\left(t_{90}\right)$ and its first derivative $E^{\prime }\left(t_{90}\right)$ and the second derivative $E^{\prime \prime }\left(t_{90}\right)$ in the interval $t_{90}\in \left[0,{1372\;}^{\circ }C\right]$ are shown in Figure 5.

**Figure 6:**
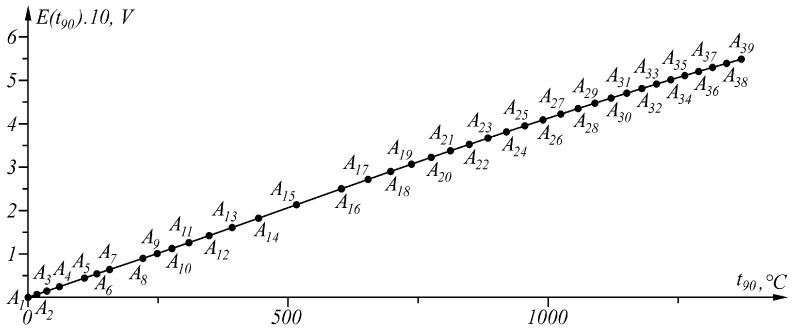
Segmentation of the characteristic in the temperature range $t_{90}\in \left[0,\;1371{.655\;}^{\circ }C\right]$.

**Figure 7:**
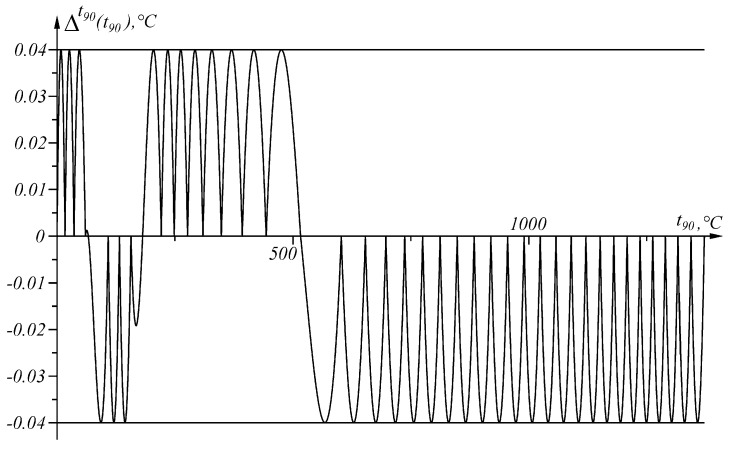
Graphical representation of the absolute error $\Delta_{i}^{t_{90}}\left(t_{90}\right)$ in the temperature range $t_{90}\in \left[0,\;1371{.655\;}^{\circ }C\right]$.

The results of applying the approach at $\left|\Delta^{t_{90}}\right|=0.04{\;}^{\circ }C$ in the temperature range $t_{90_{A_{1}}}=0{\;}^{\circ }C$, $t_{90_{A_{n}\equiv B}}=1371.655{\;}^{\circ }C$ are shown numerically in Table 1 and in Figure 6. Function $\Delta_{i}^{t_{90}}\left(t_{90}\right)$:

(8) $$\begin{array}{l} \Delta_{i}^{t_{90}}\left(t_{90}\right)=t_{90}-t_{90_{A_{i}}}-\left(E\left(t_{90}\right)-E_{A_{i}}\right)\frac{t_{90_{A_{i+1}}}-t_{90_{A_{i}}}}{E_{A_{i+1}}-E_{A_{i}}},\\ t_{90}\in \left[t_{90_{A_{i}}},t_{90_{A_{i+1}}}\right],\;i=\bar{1,n-1}. \end{array}$$

is shown in Figure 7.

Error $\Delta_{i}^{t_{90}}\left(t_{90}\right)$, defined as the difference between the actual and the measured value (8), is positive in the intervals in which the second derivative $E^{\prime \prime }\left(t_{90}\right)$ of the sensory characteristic $E\left(t_{90}\right)$ is positive, and negative in the intervals in which the second derivative $E^{\prime \prime }\left(t_{90}\right)\;$of the sensory characteristic $E\left(t_{90}\right)$ is negative (Figure 5 and Figure 7).

### 3.2. Microcontroller and LabVIEW Implementation of the Inverse Sensor Characteristics Linearization Algorithm

This design uses an Adafruit ESP32 Feather V2 [38] development board based on Espressif Systems’ ESP32 microcontroller [39]. The linearization is implemented by splitting the individual segments to ensure that the maximum predefined error requirements are satisfied. For this particular implementation, the coordinates of the points defining each interval were stored in two one-dimensional arrays each with 39 elements of type float (see Table 1). The input parameter of the implemented function is the generated voltage of the thermocouple, which must be compensated for the cold junction temperature. The variable to which its value is assigned is also of type float, with the voltage being given in µV. Following this, the determination of which of the 38 intervals the measured temperature fell into was made using the formula shown in Algorithm 1.
**Algorithm 1.** Linearization of a k-Type thermocouple**Algorithm** K-Type Linearization**Input:** Measured and compensated voltage, **Measured_Voltage**, floating point type**Output:** Calculated temperature, **Temperature**, floating point type*Initialization: Define the points determining the coordinates of each interval:**U_set[1], U_set[2]… U_set[End] and T_set[1], T_set[2]… T_set[End]*1: Determine the interval, where the measured temperature is situated If ((**Measured_Voltage** > U_set[n]) and (**Measured_Voltage** ≤ U_set[n+1]))2: Calculate the temperature by the formula**Temperature** = (Measured_Voltage − U_set[n])×((T_set[n+1] − T_set[n])/(U_set[n+1] − U_set[n])) + T_set[n];3: Return **Temperature**

The implementation of the function for the particular example considered in Table 1 took 449 bytes of Flash memory and 4 bytes of RAM, with the size optimization set to -Os. Only 312 bytes were needed to define the two arrays, out of the 449 used.

**Figure 8:**
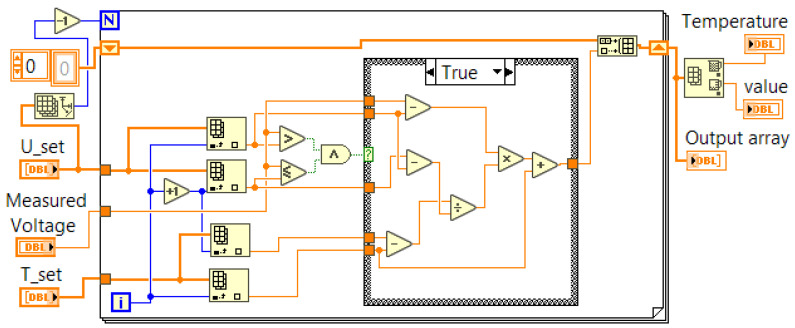
Implementation of the suggested algorithm in the graphical programming environment LabVIEW.

The algorithm was also implemented as a virtual instrument in the LabVIEW programming environment (see Figure 8). The version used by the authors to validate the proposed virtual instrument was LabVIEW 2012, but it would work on any newer version. The input parameters were the generated voltage from the thermocouple (Measured Voltage) and the two arrays with the stored coordinate values for each point (U_set and T_set). The output parameters were the converted temperature and an array (output array) in which only one element will have a value other than the constant NaN, and this will be the converted temperature. For input voltages that are in the specified range, there will be only one case where the condition of the “case” structure will be “True”. This is the case where the temperature needs to be calculated. In all other cases, the element with the constant “NaN” is added to the “Output array”.

**Figure 9:**
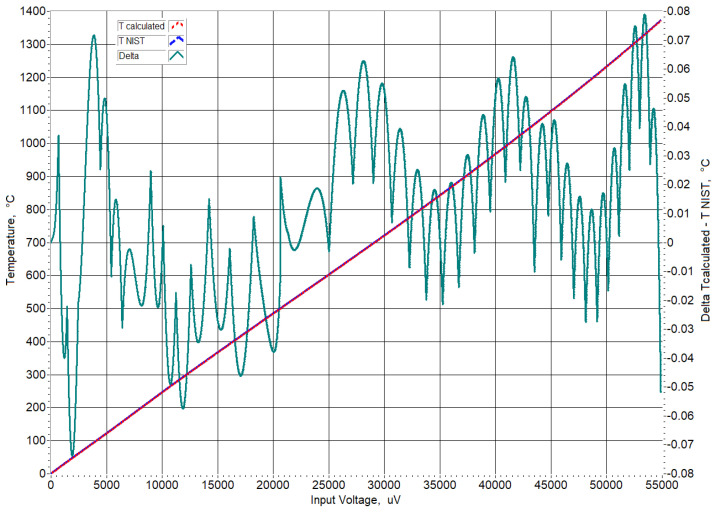
Linearized output in the range $t_{90}\in \left[0,\;1371{.655\;}^{\circ }C\right]$.

To prove the correct operation of the developed virtual instrument, values in the range 0 ÷ 54874.662 μV with step 0.01 μV were fed to its input. Figure 9 shows the linearized output, the temperature calculated by approximate inverse functions giving temperature, $t_{90}$, as a function of the thermoelectric voltage and the difference between them.

.

We observed that the difference did not exceed the sum error of the approximation and the polynomial for the corresponding temperature range (Table 2).

### 3.3. Linearization of the Inverse Characteristic of Type J Thermocouples in the Temperature Range $t_{90}\in \left[-210,760{\;}^{\circ }C\right]$

The thermoelectric voltage E in microvolts, as a function of temperature $t_{90}$ in degrees Celsius, in the range $t_{90}\in \left[-210,760^{\;\circ }C\right]$ is defined by:

(9) $$E\left(t_{90}\right)=\overset{8}{\underset{i=0}{\sum }}c_{i}\;{\left(t_{90}\right)}^{i},$$

where the coefficients $c_{i}$ are specified in NIST ITS-90 [37].

With the help of the proposed approach, the problem of interval linearization of the inverse characteristic of the sensory characteristic in question, including inflection points, will be solved.

**Figure 10:**
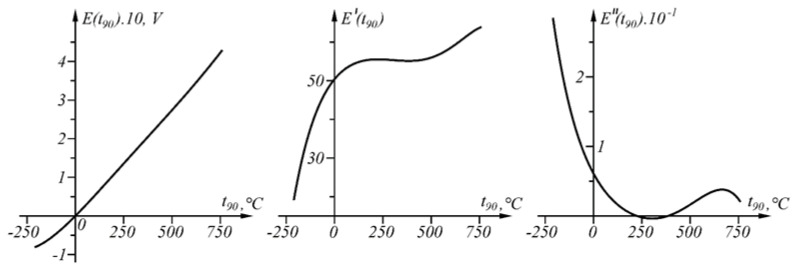
Functions $E\left(t_{90}\right)$, its first derivative $E^{\prime }\left(t_{90}\right)$ and second derivative $E^{\prime \prime }\left(t_{90}\right)$ in the interval $t_{90}\in \left[-210,760{\;}^{\circ }C\right]$.

The graphs of the function $E\left(t_{90}\right)$ and the first $E^{\prime }\left(t_{90}\right)$ and the second $E^{\prime \prime }\left(t_{90}\right)$ its derivatives in the interval $t_{90}\in \left[-210,760{\;}^{\circ }C\right]$, are shown in Figure 10.

**Figure 11:**
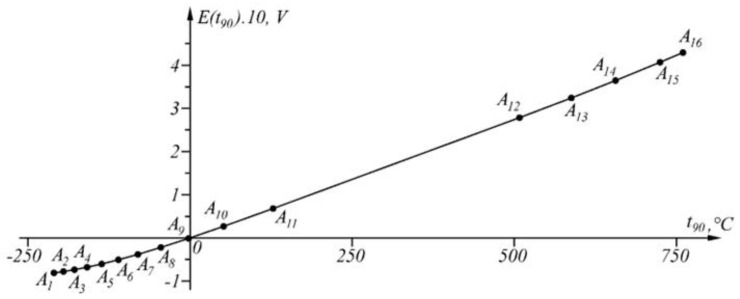
Segmentation in the temperature range $t_{90}\in \left[-210,760{\;}^{\circ }C\right]$.

**Figure 12:**
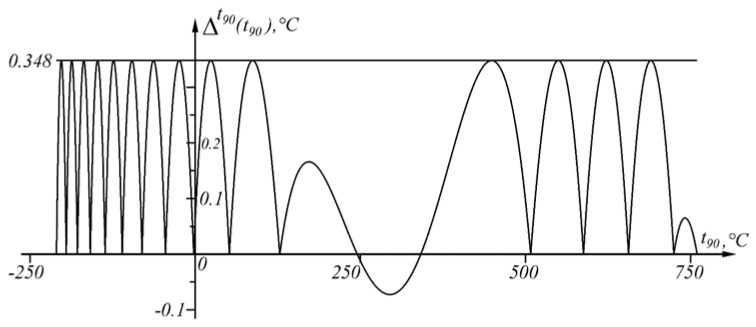
Graphical representation of the absolute error $\Delta_{i}^{t_{90}}\left(t_{90}\right)$ in the range $t_{90}\in \left[-210,760{\;}^{\circ }C\right]$.

The results of applying the approach at $\left|\Delta^{t_{90}}\right|=0.348{\;}^{\circ }C$ in the temperature range $t_{90_{A_{1}}}=-{210\;}^{\circ }C$, $t_{90_{A_{n}\equiv B}}={760\;}^{\circ }C$ are shown numerically in Table 3 and graphically in Figure 11, and the graph of the function $\Delta_{i}^{t_{90}}\left(t_{90}\right)$ is shown in Figure 12.

## 4. Conclusions

This paper presented a generalized approach for linear interval approximation of sensor characteristics $y=y\left(x\right)$, representing differentiable functions where the sign of the curvature changed, i.e., including inflection points. The goal was to obtain in a discrete form the inverse sensory characteristic in the species $x_{i}=x_{i}\left(y_{i}\right),i=\bar{1,n}$, at a pre-set maximum error $\Delta^{x}$, while minimizing the number of points determining the characteristic. This approach enabled the utilization of low-cost microcontrollers.

The approach, similar to the approach presented in [1], was characterized by the fact that when the maximum approximation error is set $\Delta^{x}$ in the linearization of the inverse sensing characteristic, the inverse sensing characteristic $x_{i}=x_{i}\left(y_{i}\right),i=\bar{1,n}\;$is found directly in linearized form. The advantages of the herein-developed approach are as follows:

- The approach is applied in intervals, and at each subsequent step (each subsequent interval), as a result of analogously solving the task under the new initial conditions, the desired solution is obtained directly, containing, in turn, the initial conditions for the next step;
- The maximum linearization error of the inverse response of the sensor in all but the last interval is the same;
- The approach makes it possible to set a different maximum predefined error bound in each subsequent interval.

The proposed generalized approach was tested in the linearization of the inverse sensor characteristic of Type K thermocouples [37]. When the maximum approximation error is set $\left|\Delta^{t_{90}}\right|=0.04{\;}^{\circ }C$ in interval $t_{90}\in \left[0,1371.655^{\;\circ }C\right]$, the inverse sensory characteristic $t_{90}=t_{90}\left(E\right)$ (representing a broken line) is described by 39 points (Figure 6).

The proposed generalized approach was also tested in the linearization of the inverse sensor characteristic of Type J thermocouples [39]. When the maximum approximation error is set $\left|\Delta^{t_{90}}\right|=0.348{\;}^{\circ }C$ in interval $t_{90}\in \left[-210,{760\;}^{\circ }C\right]$, the inverse sensor characteristic $t_{90}=t_{90}\left(E\right)$ (representing a broken line) is described by 16 points (Figure 11).

## Figures and Tables

**Table 1:** Interval linearization results in a range $t_{90}\in \left[0,\;1371.655^{\;\circ }C\right]$.

$A_{1}A_{2}$ $A_{1}\left(0,\;0.000\right)$ $A_{2}\left(16.882,\;672.556\right)$	$A_{2}A_{3}$ $A_{2}\left(16.882,\;672.556\right)$ $A_{3}\left(35.972,\;1446.874\right)$	$A_{3}A_{4}$ $A_{3}\left(35.972,\;1446.874\right)$ $A_{4}\left(60.200,\;2444.756\right)$	$A_{4}A_{5}$ $A_{4}\left(60.200,\;2444.756\right)$ $A_{5}\left(108.378,\;4442.207\right)$
$t_{90}\in \left[0,\;16.882\right]$	$t_{90}\in \left[16.882,\;35.972\right]$	$t_{90}\in \left[35.972,\;60.200\right]$	$t_{90}\in \left[60.200,\;108.378\right]$
$t_{90_{E_{1}}}=8.312$	$t_{90_{E_{2}}}=26.197$	$t_{90_{E_{3}}}=47.351$	$t_{90_{E_{4}}}=93.078$
$\Delta_{1}^{t_{90}}=0.04$	$\Delta_{2}^{t_{90}}=0.04$	$\Delta_{3}^{t_{90}}=0.04$	$\Delta_{4}^{t_{90}}=-0.04$
$A_{5}A_{6}$ $A_{5}\left(108.378,\;4442.207\right)$ $A_{6}\left(132.208,\;5418.271\right)$	$A_{6}A_{7}$ $A_{6}\left(132.208,\;5418.271\right)$ $A_{7}\left(156.708,\;6408.103\right)$	$A_{7}A_{8}$ $A_{7}\left(156.708,\;6408.103\right)$ $A_{8}\left(220.884,\;8975.453\right)$	$A_{8}A_{9}$ $A_{8}\left(220.884,\;8975.453\right)$ $A_{9}\left(248.358,\;10,086.534\right)$
$t_{90}\in \left[108.378,\;132.208\right]$	$t_{90}\in \left[132.208,\;156.708\right]$	$t_{90}\in \left[156.708,\;220.884\right]$	$t_{90}\in \left[220.884,\;248.358\right]$
$t_{90_{E_{5}}}=120.487$	$t_{90_{E_{6}}}=144.107$	$t_{90_{E_{7}}}=204.448$	$t_{90_{E_{8}}}=234.769$
$\Delta_{5}^{t_{90}}=-0.04$	$\Delta_{6}^{t_{90}}=-0.04$	$\Delta_{7}^{t_{90}}=0.04$	$\Delta_{8}^{t_{90}}=0.04$
$A_{9}A_{10}$ $A_{9}\left(248.358,\;10,086.534\right)$ $A_{10}\left(276.647,\;11,244.101\right)$	$A_{10}A_{11}$ $A_{10}\left(276.647,\;11,244.101\right)$ $A_{11}\left(309.309,\;12,594.865\right)$	$A_{11}A_{12}$ $A_{11}\left(309.309,\;12,594.865\right)$ $A_{12}\left(348.251,\;14,219.886\right)$	$A_{12}A_{13}$ $A_{12}\left(348.251,\;14,219.886\right)$ $A_{13}\left(392.599,\;16,084.688\right)$
$t_{90}\in \left[248.358,\;276.647\right]$	$t_{90}\in \left[276.647,\;309.309\right]$	$t_{90}\in \left[309.309,\;348.251\right]$	$t_{90}\in \left[348.251,\;392.599\right]$
$t_{90_{E_{9}}}=262.283$	$t_{90_{E_{10}}}=292.511$	$t_{90_{E_{11}}}=328.276$	$t_{90_{E_{12}}}=370.030$
$\Delta_{9}^{t_{90}}=0.04$	$\Delta_{10}^{t_{90}}=0.04$	$\Delta_{11}^{t_{90}}=0.04$	$\Delta_{12}^{t_{90}}=0.04$
$A_{13}A_{14}$ $A_{13}\left(392.599,\;16,084.688\right)$ $A_{14}\left(443.288,\;18,230.712\right)$	$A_{14}A_{15}$ $A_{14}\left(443.288,\;18,230.712\right)$ $A_{15}\left(516.197,\;21,334.882\right)$	$A_{15}A_{16}$ $A_{15}\left(516.197,\;21,334.882\right)$ $A_{16}\left(602.521,\;25,012.615\right)$	$A_{16}A_{17}$ $A_{16}\left(602.521,\;25,012.615\right)$ $A_{17}\left(653.510,\;27,173.147\right)$
$t_{90}\in \left[392.599,\;443.288\right]$	$t_{90}\in \left[443.288,\;516.197\right]$	$t_{90}\in \left[516.197,\;602.521\right]$	$t_{90}\in \left[602.521,\;653.510\right]$
$t_{90_{E_{13}}}=417.202$	$t_{90_{E_{14}}}=475.511$	$t_{90_{E_{15}}}=567.800$	$t_{90_{E_{16}}}=628.945$
$\Delta_{13}^{t_{90}}=0.04$	$\Delta_{14}^{t_{90}}=0.04$	$\Delta_{15}^{t_{90}}=-0.04$	$\Delta_{16}^{t_{90}}=-0.04$
$A_{17}A_{18}$ $A_{17}\left(653.510,\;27,173.147\right)$ $A_{18}\left(697.047,\;29,005.203\right)$	$A_{18}A_{19}$ $A_{18}\left(697.047,\;29,005.203\right)$ $A_{19}\left(737.039,\;30,675.189\right)$	$A_{19}A_{20}$ $A_{19}\left(737.039,\;30,675.189\right)$ $A_{20}\left(775.070,\;32,250.248\right)$	$A_{20}A_{21}$ $A_{20}\left(775.070,\;32,250.248\right)$ $A_{21}\left(811.974,\;33,765.632\right)$
$t_{90}\in \left[653.510,\;697.047\right]$	$t_{90}\in \left[697.047,\;737.039\right]$	$t_{90}\in \left[737.039,\;775.070\right]$	$t_{90}\in \left[775.070,\;811.974\right]$
$t_{90_{E_{17}}}=675.665$	$t_{90_{E_{18}}}=717.243$	$t_{90_{E_{19}}}=756.163$	$t_{90_{E_{20}}}=793.579$
$\Delta_{17}^{t_{90}}=-0.04$	$\Delta_{18}^{t_{90}}=-0.04$	$\Delta_{19}^{t_{90}}=-0.04$	$\Delta_{20}^{t_{90}}=-0.04$
$A_{21}A_{22}$ $A_{21}\left(811.974,\;33,765.632\right)$ $A_{22}\left(848.239,\;35,241.740\right)$	$A_{22}A_{23}$ $A_{22}\left(848.239,\;35,241.740\right)$ $A_{23}\left(884.134,\;36,689.927\right)$	$A_{23}A_{24}$ $A_{23}\left(884.134,\;36,689.927\right)$ $A_{24}\left(919.767,\;38,114.730\right)$	$A_{24}A_{25}$ $A_{24}\left(919.767,\;38,114.730\right)$ $A_{25}\left(955.104,\;39,514.991\right)$
$t_{90}\in \left[811.974,\;848.239\right]$	$t_{90}\in \left[848.239,\;884.134\right]$	$t_{90}\in \left[884.134,\;919.767\right]$	$t_{90}\in \left[919.767,\;955.104\right]$
$t_{90_{E_{21}}}=830.133$	$t_{90_{E_{22}}}=866.197$	$t_{90_{E_{23}}}=901.959$	$t_{90_{E_{24}}}=937.452$
$\Delta_{21}^{t_{90}}=-0.04$	$\Delta_{22}^{t_{90}}=-0.04$	$\Delta_{23}^{t_{90}}=-0.04$	$\Delta_{24}^{t_{90}}=-0.04$
$A_{25}A_{26}$ $A_{25}\left(955.104,\;39,514.991\right)$ $A_{26}\left(989.996,\;40,885.113\right)$	$A_{26}A_{27}$ $A_{26}\left(989.996,\;40,885.113\right)$ $A_{27}\left(1024.231,\;42,217.028\right)$	$A_{27}A_{28}$ $A_{27}\left(1024.231,\;42,217.028\right)$ $A_{28}\left(1057.590,\;43,502.686\right)$	$A_{28}A_{29}$ $A_{28}\left(1057.590,\;43,502.686\right)$ $A_{29}\left(1089.912,\;44,736.246\right)$
$t_{90}\in \left[955.104,\;989.996\right]$	$t_{90}\in \left[989.996,\;1024.231\right]$	$t_{90}\in \left[1024.231,\;1057.590\right]$	$t_{90}\in \left[1057.590,\;1089.912\right]$
$t_{90_{E_{25}}}=972.582$	$t_{90_{E_{26}}}=1007.164$	$t_{90_{E_{27}}}=1040.978$	$t_{90_{E_{28}}}=1073.829$
$\Delta_{25}^{t_{90}}=-0.04$	$\Delta_{26}^{t_{90}}=-0.04$	$\Delta_{27}^{t_{90}}=-0.04$	$\Delta_{28}^{t_{90}}=-0.04$
$A_{29}A_{30}$ $A_{29}\left(1089.912,\;44,736.246\right)$ $A_{30}\left(1121.115,\;45,915.218\right)$	$A_{30}A_{31}$ $A_{30}\left(1121.115,\;45,915.218\right)$ $A_{31}\left(1151.210,\;47,040.472\right)$	$A_{31}A_{32}$ $A_{31}\left(1151.210,\;47,040.472\right)$ $A_{32}\left(1180.276,\;48,115.572\right)$	$A_{32}A_{33}$ $A_{32}\left(1180.276,\;48,115.572\right)$ $A_{33}\left(1208.447,\;49,145.979\right)$
$t_{90}\in \left[1089.912,\;1121.115\right]$	$t_{90}\in \left[1121.115,\;1151.210\right]$	$t_{90}\in \left[1151.210,\;1180.276\right]$	$t_{90}\in \left[1180.276,\;1208.447\right]$
$t_{90_{E_{29}}}=1105.594$	$t_{90_{E_{30}}}=1136.239$	$t_{90_{E_{31}}}=1165.810$	$t_{90_{E_{32}}}=1194.416$
$\Delta_{29}^{t_{90}}=-0.04$	$\Delta_{30}^{t_{90}}=-0.04$	$\Delta_{31}^{t_{90}}=-0.04$	$\Delta_{32}^{t_{90}}=-0.04$
$A_{33}A_{34}$ $A_{33}\left(1208.447,\;49,145.979\right)$ $A_{34}\left(1235.896,\;50,138.464\right)$	$A_{34}A_{35}$ $A_{34}\left(1235.896,\;50,138.464\right)$ $A_{35}\left(1262.828,\;51,100.866\right)$	$A_{35}A_{36}$ $A_{35}\left(1262.828,\;51,100.866\right)$ $A_{36}\left(1289.490,\;52,042.271\right)$	$A_{36}A_{37}$ $A_{36}\left(1289.490,\;52,042.271\right)$ $A_{37}\left(1316.189,\;52,973.750\right)$
$t_{90}\in \left[1208.447,\;1235.896\right]$	$t_{90}\in \left[1235.896,\;1262.828\right]$	$t_{90}\in \left[1262.828,\;1289.490\right]$	$t_{90}\in \left[1289.490,\;1316.189\right]$
$t_{90_{E_{33}}}=1222.211$	$t_{90_{E_{34}}}=1249.382$	$t_{90_{E_{35}}}=1276.157$	$t_{90_{E_{36}}}=1302.808$
$\Delta_{33}^{t_{90}}=-0.04$	$\Delta_{34}^{t_{90}}=-0.04$	$\Delta_{35}^{t_{90}}=-0.04$	$\Delta_{36}^{t_{90}}=-0.04$
$A_{37}A_{38}$ $A_{37}\left(1316.189,\;52,973.750\right)$ $A_{38}\left(1343.352,\;53,910.167\right)$	$A_{38}A_{39}$ $A_{38}\left(1343.352,\;53,910.167\right)$ $A_{39}\left(1371.655,\;54,874.662\right)$		
$t_{90}\in \left[1316.189,\;1343.352\right]$	$t_{90}\in \left[1343.352,\;1371.655\right]$		
$t_{90_{E_{37}}}=1329.696$	$t_{90_{E_{38}}}=1357.354$		
$\Delta_{37}^{t_{90}}=-0.04$	$\Delta_{38}^{t_{90}}=-0.04$		

**Table 2:** Error range for the temperature, t90, as a function of the thermoelectric voltage, in selected temperature and voltage ranges [37].

Temperature Range:	−200 to 0 °C	0 to 500 °C	500 to 1 372 °C
**Voltage Range:**	−5891 to 0 μV	0 to 20,644 μV	20 644 to 54,886 μV
**Error Range:**	0.04 °C to −0.02 °C	0.04 °C to −0.05 °C	0.06 °C to −0.05 °C

**Table 3:** Interval linearization results in the range $t_{90}\in \left[-210,760{\;}^{\circ }C\right]$.

$A_{1}A_{2}$ $A_{1}\left(-210,\;-8095.380\right)$ $A_{2}\left(-195.274,\;-7784.257\right)$	$A_{2}A_{3}$ $A_{2}\left(-195.274,\;-7784.257\right)$ $A_{3}\left(-178.302,\;-7356.617\right)$	$A_{3}A_{4}$ $A_{3}\left(-178.302,\;-7356.617\right)$ $A_{4}\left(-158.798,\;-6783.821\right)$	$A_{4}A_{5}$ $A_{4}\left(-158.798,\;-6783.821\right)$ $A_{5}\left(-136.378,\;-6031.463\right)$
$t_{90}\in \left[-210,\;-195.274\right]$	$t_{90}\in \left[-195.274,\;-178.302\right]$	$t_{90}\in \left[-178.302,\;-158.798\right]$	$t_{90}\in \left[-158.798,\;-136.378\right]$
$t_{90_{E_{1}}}=-202.698$	$t_{90_{E_{2}}}=-186.870$	$t_{90_{E_{3}}}=-168.660$	$t_{90_{E_{4}}}=-147.735$
$\Delta_{1}^{t_{90}}=0.348$	$\Delta_{2}^{t_{90}}=0.348$	$\Delta_{3}^{t_{90}}=0.348$	$\Delta_{4}^{t_{90}}=0.348$
$A_{5}A_{6}$ $A_{5}\left(-136.378,\;-6031.463\right)$ $A_{6}\left(-110.511,\;-5056.892\right)$	$A_{6}A_{7}$ $A_{6}\left(-110.511,\;-5056.892\right)$ $A_{7}\left(-80.438,\;-3804.623\right)$	$A_{7}A_{8}$ $A_{7}\left(-80.438,\;-3804.623\right)$ $A_{8}\left(-45.000,\;-2197.052\right)$	$A_{8}A_{9}$ $A_{8}\left(-45.000,\;-2197.052\right)$ $A_{9}\left(-2.242,\;-112.816\right)$
$t_{90}\in \left[-136.378,\;-110.511\right]$	$t_{90}\in \left[-110.511,\;-80.438\right]$	$t_{90}\in \left[-80.438,\;-45.000\right]$	$t_{90}\in \left[-45.000,\;-2.242\right]$
$t_{90_{E_{5}}}=-123.643$	$t_{90_{E_{6}}}=-95.749$	$t_{90_{E_{7}}}=-63.115$	$t_{90_{E_{8}}}=-24.233$
$\Delta_{5}^{t_{90}}=0.348$	$\Delta_{6}^{t_{90}}=0.348$	$\Delta_{7}^{t_{90}}=0.348$	$\Delta_{8}^{t_{90}}=0.348$
$A_{9}A_{10}$ $A_{9}\left(-2.242,\;-112.816\right)$ $A_{10}\left(51.729,\;2676.741\right)$	$A_{10}A_{11}$ $A_{10}\left(51.729,\;2676.741\right)$ $A_{11}\left(128.045,\;6801.389\right)$	$A_{11}A_{12}$ $A_{11}\left(128.045,\;6801.389\right)$ $A_{12}\left(507.969,\;27,839.325\right)$	$A_{12}A_{13}$ $A_{12}\left(507.969,\;27,839.325\right)$ $A_{13}\left(587.888,\;32,396.359\right)$
$t_{90}\in \left[-2.242,\;51.729\right]$	$t_{90}\in \left[51.729,\;128.045\right]$	$t_{90}\in \left[128.045,\;507.969\right]$	$t_{90}\in \left[507.969,\;587.888\right]$
$t_{90_{E_{9}}}=23.657$	$t_{90_{E_{10}}}=87.116$	$t_{90_{E_{11}}}=448.839$	$t_{90_{E_{12}}}=549.777$
$\Delta_{9}^{t_{90}}=0.348$	$\Delta_{10}^{t_{90}}=0.348$	$\Delta_{11}^{t_{90}}=0.348$	$\Delta_{12}^{t_{90}}=0.348$
$A_{13}A_{14}$ $A_{13}\left(587.888,\;32,396.359\right)$ $A_{14}\left(656.077,\;36,437.746\right)$	$A_{14}A_{15}$ $A_{14}\left(656.077,\;36,437.746\right)$ $A_{15}\left(724.711,\;40,678.242\right)$	$A_{15}A_{16}$ $A_{15}\left(724.711,\;40,678.242\right)$ $A_{16}\left(760,\;42,918.641\right)$	
$t_{90}\in \left[587.888,\;656.077\right]$	$t_{90}\in \left[656.077,\;724.711\right]$	$t_{90}\in \left[724.711,\;760\right]$	
$t_{90_{E_{13}}}=622.514$	$t_{90_{E_{14}}}=689.931$	$t_{90_{E_{15}}}=741.725$	
$\Delta_{13}^{t_{90}}=0.348$	$\Delta_{14}^{t_{90}}=0.348$	$\Delta_{15}^{t_{90}}=0.065$	

## Data Availability

Data are contained within the article.
